# PAACC: A MINDFULNESS-BASED MULTICOMPONENT PROGRAM FOR FAMILY CAREGIVERS OF PERSONS WITH DEMENTIA

**DOI:** 10.1093/geroni/igz038.1061

**Published:** 2019-11-08

**Authors:** Lauren Hagemann, Katherine Luci, Mamta Sapra, Jyoti Savla, Lindsey Jacobs, Tonda Yates

**Affiliations:** 1 Salem VA Medical Center, Salem, Virginia, United States; 2 Virginia Tech, Blacksburg, Virginia, United States; 3 VA Boston Healthcare System, Brockton, Massachusetts, United States

## Abstract

Few interventions for family caregivers of persons with dementia (PwD) focus on both dementia care skill-building and the enhancement of acceptance and compassion towards oneself and the PwD. We designed a multicomponent, mindfulness-based 4-session caregiver intervention (Practice of Acceptance, Awareness, and Compassion in Caregiving, or “PAACC”) to reduce burden in caregivers of family members with Alzheimer’s disease and related dementias (AD/ADRD) and TBI-related AD. A prospective, randomized trial design is being implemented to compare the effectiveness of PAACC to the well-known, evidence-based REACH-VA intervention. Seventeen family caregivers (Mean Age = 68.71 years; 82% women; 30% had high school or less education) have participated in the trial thus far and provided qualitative responses to acceptability questions. High acceptability was noted for all intervention components of PAACC. Participants completed 95% of mindfulness homework during the study period. A majority also reported practicing spontaneous, informal mindfulness while engaged in daily activities (e.g., going for a walk, cooking). One participant noted incorporating mindfulness in her daily spiritual practice. The majority remarked that PAACC taught them to be more aware and accepting of the PwD’s illness. Others mentioned becoming more aware of which stressors triggered them, and that they were able to avoid arguments with the PwD because of this increased awareness. Overall, our results suggest that this mindfulness-based multicomponent intervention is a promising method for promoting stress reduction for family caregivers of persons with dementia regardless of age, stage of dementia, education level, or rurality. Implications for research and practice will be discussed.

